# Considerations on the Current Universal Vaccination Policy against Hepatitis A in Greece after Recent Outbreaks

**DOI:** 10.1371/journal.pone.0116939

**Published:** 2015-01-15

**Authors:** Kassiani Mellou, Theologia Sideroglou, Vassiliki Papaevangelou, Anna Katsiaflaka, Nikolaos Bitsolas, Eleni Verykouki, Eleni Triantafillou, Agoritsa Baka, Theano Georgakopoulou, Christos Hadjichristodoulou

**Affiliations:** 1 Hellenic Centre for Disease Control and Prevention, Athens, Greece; 2 Third Department of Pediatrics, University of Athens, General University Hospital “ATTIKON”, Chaidari, Athens, Greece; 3 Department of hygiene and epidemiology, Medical Faculty, University of Thessaly, Larissa, Greece; National Institute for Viral Disease Control and Prevention, CDC, China, CHINA

## Abstract

Greece is the only European Union member state that in 2008 included hepatitis A (HAV) vaccine in the routine national childhood immunization program (NCIP). Given that the resources allocated to public health have dramatically decreased since 2008 and that Greece is a low endemicity country for the disease, the benefit from universal vaccination has been questioned. The aim of this paper is to summarize the available epidemiological data of the disease for 1982-2013, and discuss the effects of universal vaccination on disease morbidity. Descriptive analysis, ARIMA modeling and time series intervention analysis were conducted using surveillance data of acute HAV. A decreasing trend of HAV notification rate over the years was identified (p<0.001). However, universal vaccination (~ 80% vaccine coverage of children) had no significant effect on the annual number of reported cases (p = 0.261) and has resulted to a progressive increase of the average age of infection in the general population. The mean age of cases before the inclusion of the vaccine to NCIP (24.1 years, SD = 1.5) was significantly lower than the mean age of cases after 2008 (31.7 years, SD = 2.1) (p<0.001). In the last decade, one third of all reported cases were Roma (a population accounting for 1.5% of the country’s total population) and in 2013 three outbreaks with 16, 9 and 25 Roma cases respectively, were recorded, indicating the decreased effectiveness of the current immunization strategy in this group. Data suggest that universal vaccination may need to be re-considered. Probably a more cost effective approach would be to implement a program that will include: a) vaccination of high risk groups, b) universal vaccination of Roma children and improving conditions at Roma camps, c) education of the population and travel advice, and d) enhancement of the control measures to increase safety of shellfish and other foods.

## Introduction

Infection with Hepatitis A Virus (HAV) is an acute, self-limited disease of the liver, with worldwide distribution [1]. The infection is usually asymptomatic among children aged less than six years, while among older children and adults it leads to clinical disease with jaundice in more than 70% of the cases [1]. Geographical areas are characterized by high, intermediate, low or very low levels of endemicity [2–4].

The efficacy and effectiveness of the available vaccines against HAV is high and vaccination provides long-term protection [3, 5, 6]. Still, the appropriate role of vaccination programs, especially of universal vaccination strategies remains ambiguous [7, 8]. Universal childhood vaccination is recommended by the World Health Organization on the basis of acute HAV incidence, change in the endemicity from high to intermediate, and cost-effectiveness [3]. Studies have demonstrated a strong relationship between the infection risk and the vaccination cost-effectiveness [9]. Published cost-effectiveness analyses of universal vaccination resulted in cost-savings in high incidence regions [9–12].

The infection risk is directly linked to the prevalence of anti-HAV by age group in a population and, thus, defines the appropriate vaccination policy in the population [3–4]. The availability of seroprevalence data by age enables indirect measurement of age-specific HAV incidence rates, and is considered as the best way to describe the epidemiology of the disease in a country [3, 13].

In highly endemic areas (>90% of the population become immune by the age of 10), infection is usually acquired in early childhood as an asymptomatic or mild infection. In these areas, there are almost no susceptible adolescents and adults, and the notification rate is low [3, 4, 13]. Consequently, universal vaccination of children is not recommended [3, 4]. In communities with intermediate endemicity (>50% of the population are immune by the age of 15 years, with <90% of infections by the age of 10), the incidence is high enough to yield significant risk of infection among adolescents and adults [3, 4, 13]. In these areas, HAV burden is increasing and universal vaccination programs should be considered [3, 4]. In low (>50% till 30 years, with <50% by the age of 15) and very low (<50% immunity among persons older than 30 years of age) endemic countries, disease may occur among specific risk groups, such as travelers and intravenous drug users, or after the consumption of a specific product (as in the recent outbreaks in Europe) and targeted vaccination of high-risk groups rather than universal vaccination is usually recommended [3, 4].

Even though universal vaccination has not been broadly adopted in Europe, it has been successfully implemented, in heterogeneous communities with consistently elevated HAV rates with respect to the national average, in Puglia in Italy [14], in Catalonia and other areas of Spain [14, 15], as well as in non-European countries [16–18].

In Greece, HAV vaccine was first licensed in 1999. Until 2008, vaccination was recommended for high risk groups of the population. However, pediatricians advised vaccination of children on parents’ expense and in 2006 the vaccine coverage of the 6-year old children was estimated at 37% (for the two doses of the vaccine) [19].

In 2008, the vaccine was introduced to the routine national childhood immunization program (NCIP) for all children older than 12 months and is, therefore, fully reimbursed. Given that, the public health priorities have substantially changed in the country since 2008 (e.g. West Nile Virus emergence in Greece, a number of autochthonous malaria cases) and resources allocated to public health have dramatically decreased due to the economic crisis, the benefit from universal HAV vaccination has been questioned.

The aim of this paper is to summarize the available epidemiological data of the disease in the country for the period 1982–2013 and discuss the effects of the implemented universal vaccination strategy on disease morbidity to date.

## Methods

### Hepatitis A surveillance 1982–2013

Between 1982 and 1997, aggregated HAV data were notified from the peripheral public health authorities to the Hellenic Ministry of Health. Data on age, gender or other risk factors were not collected. Since 1998, the national Mandatory Notification System (MNS) has been operating under the auspices of the newly founded Hellenic Center for Disease Control and Prevention (HCDCP). Notification forms were designed for all notifiable diseases. In 2003, in the context of harmonizing the national surveillance system with the European Union (EU) surveillance framework and of preparing for hosting the 2004 Olympic Games, the notification system was reformed. The notification time frame changed from a monthly to a weekly basis and the notification form was redesigned to include additional information, such as the place of residence, and vaccination status. It is also recorded whether the case belongs to one of the following population groups: a) travelers to an endemic country during the incubation period, b) Roma people, c) immigrants (along with the country of origin), and d) Greek Muslims living in the northern part of the country (population group considered a high risk group for HAV in Greece).

Currently, the local health care units notify new cases of HAV or clusters of cases to local public health authorities and HCDCP simultaneously. Reported cases are classified in accordance with the European case definition [20] and data are recorded in an EpiData database at HCDCP. For all recorded cases, telephone contacts with treating physicians for data validation and with the public health directorate for acquiring information regarding the case investigation (e.g. other cases in the same population, epidemiological links) are performed by HCDCP epidemiologists. National data are analyzed at a central level on a weekly basis for the detection of clusters of cases or outbreaks.

### Statistical analysis

Only laboratory confirmed cases (cases with HAV specific antibody response) were included in the statistical analysis of the available epidemiological data.

Analysis was carried out with STATA version 12 software (Stata Corporation LP, Texas, USA) and R v3.0.2 statistical software (R Foundation for Statistical Computing, Vienna, Austria). All incidences were calculated using the estimated population of Greece in the middle of each year (30th of June), according to the National Statistical Authority.

Autoregressive integrated moving average (ARIMA) modeling was performed to analyze the monthly HAV cases for the years 1982–2013 and geographical distribution of notified cases was depicted for consecutive 5-year intervals.

Data on risk factors were analyzed for the years after 2003, that the respective information was systematically collected. The annual mean number of reported cases in the general population and among Roma was calculated for each one of the 51 prefectures of the country and the non-parametric Spearman’s rank correlation coefficient (Spearman’s rho) was used to assess the association between the two means.

The identified outbreaks in 2013 were described in terms of time, place, and persons’ characteristics and the results of the investigation were summarized. Viral nucleic acids were extracted from faecal samples from outbreak cases using the QIAamp Viral RNA Mini kit (Qiagen) according to the manufacturer’s instructions and sequencing (ABI 3730 DNA Analyser) for the molecular typing of the detected viral agents was performed.

Time series intervention analysis was used to examine whether the release of HAV vaccine in the market in 1999, as well as its introduction to the routine NCIP in 2008, had an impact on the number of reported cases. Intervention analysis is used to assess the effect of an event on the time series of interest [21]. Comparison of the mean age of people with HAV infection before (2004–2008) and after the introduction of the vaccine to the NCIP (2009–2013) was conducted using the independent samples t-test.

P values less than 0.05 were considered statistically significant.

## Results

**Figure 1:**
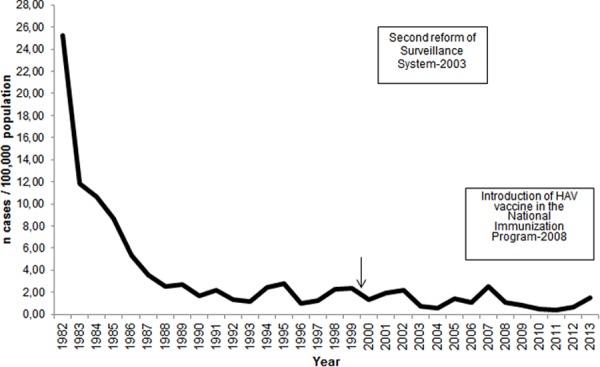
Yearly distribution of hepatitis A notification rate (cases per 100,000 population), Mandatory Notification System, Greece, 1982–2013.

ARIMA analysis revealed a decreasing trend of HAV notification rate in Greece over the years (p<0.001). The notification rate substantially dropped during the 80’s and has been quite stable ever since (Fig. 1).

**Figure 2:**
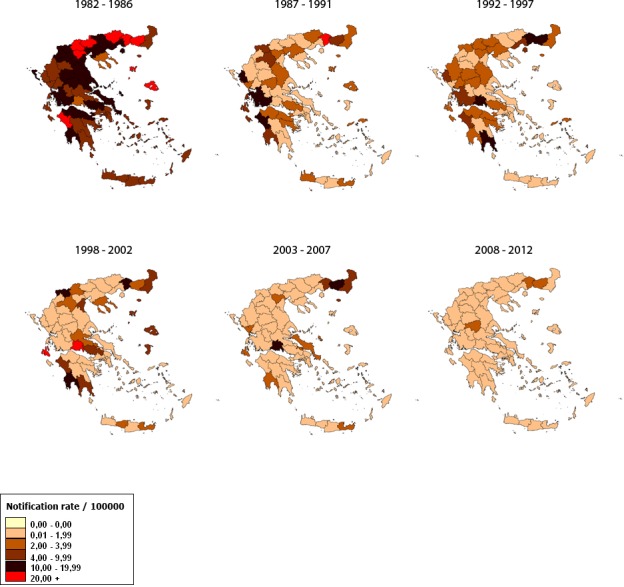
Geographical distribution of hepatitis A reported cases, Mandatory Notification System, Greece, 1982–2012 (5-year time intervals with the exception of 1992–1997 because of the following change of the system).

During 2004–2012, 995 HAV cases were reported (mean annual number of cases: 111, SD: 75). Thirty-one (3.1%) had been vaccinated against HAV (data on the number of doses are not available). The notification rate of the disease was higher in Eastern Macedonia and Thrace and in particular in the regional units of Xanthi, Rodopi and Evros (Fig. 2).

Almost one third of all reported cases (29.6%) were Roma, 96 (9.7%) cases were travel-related, 55 (9.1%) were immigrants, and 36 (3.6%) were Greek Muslims. The median age of Roma cases (5.9 years, IQR: 3.3–8.0) was significantly lower than the median age of cases in the general population (26.0 years, IQR: 17.0–35.0) (p<0.001). A statistically significant positive correlation between the number of cases among Roma and the number of cases in the general population, by prefecture of residence, was identified (Spearman’s rho = 0.704, p<0.001). In 2007, an outbreak with 139 identified cases was recorded in Eastern Macedonia and Thrace [22]. The majority of outbreak cases (82%) were Roma [22].

**Figure 3:**
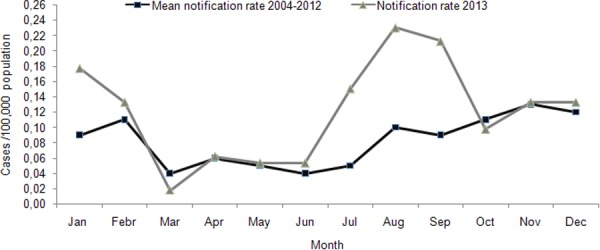
Mean monthly notification rate of hepatitis A for 2004–2012 and monthly notification rate in 2013, Mandatory Notification System, Greece.

**Figure 4:**
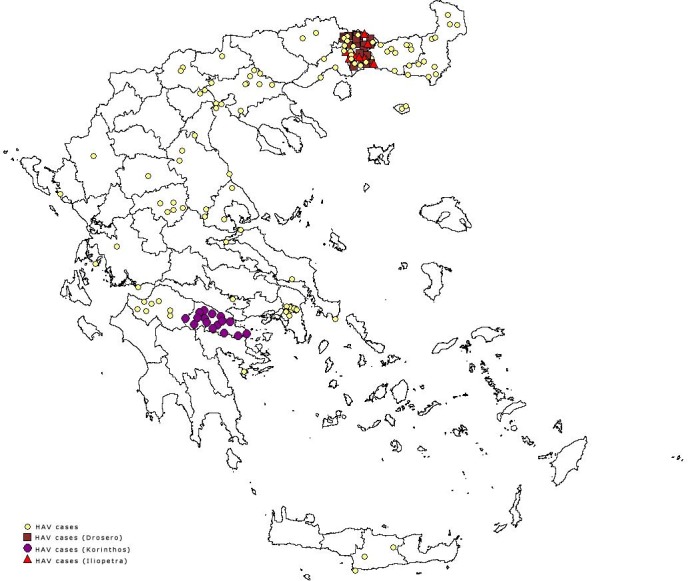
Distribution of reported outbreak and non-outbreak cases by prefecture of residence, Mandatory Notification System, Greece, 2013.

In 2013, in total 164 HAV cases were reported; 101 (61.6%) of them were Roma. For the general population, the reported number was lower than the mean annual number of reported cases for 2004–2012 (mean: 61, SD: 37.3). Among Roma, the number of reported cases was three times higher than the mean annual number of cases for 2004–2012 (mean: 33, SD: 34.2). The number of notifications increased during the first two months of 2013, as well as from July to September (Fig. 3). In the beginning of 2013 a cluster of 16 cases (median age: 7, IQR: 3–8 years) was identified in the prefecture of Korinthos in Southern Greece with the descriptive data suggesting person to person transmission. A larger outbreak with 25 Roma cases was recorded in the region of Xanthi in Drosero camp (median age 6, IQR: 3.6–7.0 years) later the same year and finally, another nine cases were recorded in the same region in a Roma camp in Iliopetra (median age: 19, IQR: 17–26 years). An epidemiological link between the two last clusters was not established. No community cases related to these outbreaks were identified. The geographical distribution of outbreak and of non-outbreak cases by prefecture of residence is given in Fig. 4. From the three faecal samples collected at Drosero, HAV viruses of genotype I, subgenotype IA were identified. The local public health authorities identified factors contributing to the occurrence of the outbreaks, such as the low vaccination coverage of the population, the low socioeconomic conditions and the poor hygiene, including inadequate sewage systems. Vaccination of the children living at the specific camps could not be performed due to lack of resources.

Intervention analysis for the years 1990–2013 showed no significant effect of the release of HAV vaccine (p = 0.328) or its introduction to the routine NCIP (p = 0.261) on the annual number of reported cases. Independent samples t-test did not reveal a significant difference in the mean age of Roma people with HAV infection after the introduction of the vaccine to NCIP in 2008 (p = 0.964). However, in the general population the mean age of cases before 2008 was significantly lower (24.1 years, SD = 1.5) than the mean age of cases after 2008 (31.7 years, SD = 2.1) (p<0.001).

## Discussion

The notification rate of HAV infection substantially declined in Greece during the 80’s and has been quite stable ever since. This decrease is attributed to the improvement of the socioeconomic conditions in the 80’s [23, 24]. In 2008, the vaccine against HAV was included in the NCIP. The decision was not based on a cost effective analysis neither on the age-specific prevalence of antibodies against HAV at that moment since national seroprevalence data were not available. Even though there have been seroprevalence studies before and after the inclusion of the vaccine to NCIP, they focused on certain population groups (children, Roma people, waste handlers, young adults joining the army, etc.) or were conducted at specific geographical areas of the country [25–29]. The World Health Organization, based on a recent effort to estimate the global prevalence of HAV infection and susceptibility, and after taking into account all the available data, has ranked Greece among the low endemicity countries for the disease [30, 31]. This questions the decision of adopting a universal strategy as opposed to the other European Union member states with low endemicity, especially in the context of the recent limitation of resources allocated to public health due to the economic crisis and the emergence of new public health priorities in the country. A national age-specific seroprevalence study, as well as an in depth cost effectiveness analysis of the current strategy are lacking.

Our data showed that the notification rate in the general population, as well as among immigrants and travelers has not significantly changed since 2008. This is in accordance with the results of a study in a large tertiary pediatric center in Athens (1999–2011), in which the overall HAV admission rates did not significantly change after 2008 [32].

In addition, introduction of HAV vaccine to the NCIP has resulted to a progressive increase of the average age of infection. The association of the routine vaccination of children with increased age of infection is well established in the literature [33] and can potentially lead to more severe cases in the general population and to a consequent increased cost for the health care system and the society, in terms of workplace absenteeism and lost productivity [4, 34].

Furthermore, the number of recorded cases in 2013 was increased and universal childhood vaccination did not prevent the recent outbreaks at Roma camps, indicating the decreased effectiveness of the current immunization strategy in this group. On the other hand, universal vaccination has probably contributed to the confinement of the recorded outbreaks and to the prevention of other community outbreaks in the recent years.

The Roma, accounting for around 1.5% of the country’s population, remains the main high risk group in the country. In our study, a geographical correlation of the occurrence of cases among Roma with the occurrence of community cases in the general population was identified. The role of Roma in the transmission of vaccine-preventable diseases in the community has been documented in the context of recent outbreaks in Greece [35] and in other countries [36, 37]. By using this information one may support that universal vaccination has probably prevented a large number of cases in the general population transmitted from Roma population. While this is may be partially true, we must keep in mind that Roma is a relatively closed population and they do not usually have much interaction with the general population.

Currently, there is no routine nationwide vaccination registry in Greece and therefore age-specific vaccination coverage data are not systematically collected. In a 2012 national study, 88% of the 6-year old children had been vaccinated with one dose and 82% with two doses of the HAV vaccine [38], while the vaccination coverage of the 3-year old children in a national study in 2013 was estimated at 80% for one dose and at 42% for two doses of the vaccine [39]. Vaccination coverage is similar to other countries that have introduced universal HAV vaccination [40]. Τhe minimum vaccination coverage necessary to stop direct person to person transmission was calculated to range from 44.5% to 63.0% in a recent study in Puglia region in Italy [33]. Roma childhood population in Greece found with 25% and 13% vaccination coverage for one and two doses of the vaccine, respectively, in a recent study [38]. Thus, it is obvious that Roma children are not vaccinated at the recommended level to inhibit person to person transmission. An important restriction for strengthening vaccination among Roma is that it is a population hard to target since they often travel within the country, almost 50% of them live in improvised constructions and hovels, while their beliefs may also affect their vaccination coverage rate [41, 42]. Moreover, additional measures for this population group would severely increase the cost of the current vaccination program.

The overall annual cost of universal vaccination can be roughly estimated at eight million Euros, based on the current annual number of births in Greece, the cost of the vaccine (two doses), the vaccination service cost and the vaccination coverage of the population. Therefore, one might even postulate that given the cost limitations and the low notification rate of the disease in the general population, a probably more cost effective approach would be to design a prevention plan that would include: a) vaccination of high risk groups of the population (immigrants, homosexuals, waste collectors etc.) as in other European Union member states, b) universal vaccination of Roma children against HAV, as part of a targeted program against all vaccine preventable diseases early in life, catch-up vaccination for susceptible adolescents, along with improving conditions at Roma camps, c) education of the general population on HAV modes of transmission and travel advice and, d) enhanced control measures to increase safety of imported foods and shellfish sold in the market. If such a change in the vaccination policy was elected, an important amount of the resources spent for universal vaccination would be invested in the above multi-dimensional program. However, one limitation would be the difficulties in vaccinating high risk groups of the population since high vaccine coverage in these groups could only be achieved through specific interventions which again are costing.

In any case, the recent limitation of the resources allocated to public health care sector in Greece, calls for different prioritization of public health needs and the current universal vaccination program against HAV may need to be re-considered.
